# Efficacy and safety of lenalidomide in diffuse large B-cell lymphoma: a meta-analysis of randomized controlled trials

**DOI:** 10.1007/s10238-022-00920-2

**Published:** 2022-10-31

**Authors:** Jia Liu, Ruihua Mi, Lin Chen, Xiaoli Guo, Taotao Liang, Qingsong Yin

**Affiliations:** The Affiliated Cancer Hospital of Zhengzhou University and Henan Cancer Hospital, Zhengzhou, China

**Keywords:** Lenalidomide, Diffuse large B-cell lymphoma, Meta-analysis, Clinical trials

## Abstract

As an immunomodulatory agent with antitumor activity, lenalidomide has been evaluated for its value in diffuse large B-cell lymphoma (DLBCL). We performed a meta-analysis to gain a better understanding of the efficacy and safety of lenalidomide in DLBCL. PubMed, Cochrane Library, and Embase were searched up to March 2022 for potential studies. The pooled hazard ratio (HR) and relative risk (RR) with 95% confidence interval (CI) were estimated by the fixed/random effects model. Overall, 6 randomized controlled trials including 1938 patients were included. The complete response rate (CRR) of the group containing lenalidomide was 47.7% (95%CI 28.5–67.2%), which was higher than the 37.8% (95%CI 16.7–61.5%) of the control group without lenalidomide (RR = 1.11, 95%CI 1.03–1.20, *P* = 0.008). The overall estimation of survival showed a benefit for progression-free survival (PFS) (HR = 0.77, 95%CI 0.66–0.90, *P* = 0.001) but not overall survival (OS) or event-free survival (EFS). The lenalidomide group had a significant incidence of grade ≥ 3 hematological adverse events (AEs) involving neutropenia (RR = 1.56, 95%CI 1.15–2.11, *P* = 0.004) and febrile neutropenia (RR = 1.81, 95%CI 1.31–2.49, *P* < 0.001), with the incidence of neutropenia (48.3%, 95%CI 37.5–59.1%) being highest. In conclusion, addition of lenalidomide results in a higher CRR and better PFS but a higher incidence of grade ≥ 3 hematological AEs involving neutropenia and febrile neutropenia.

## Introduction

Diffuse large B-cell lymphoma (DLCBL) is the most common, aggressive non-Hodgkin lymphoma (NHL) subtype, comprising approximately 30–40% of cases [1]. The disease is a highly heterogeneous lymphoma characterized by diffuse structure, mature B-cell phenotype, and cell morphology, with multiple subtypes and genetic profiles. DLCBL is divided according to the Hans classification into a germinal center type (GCB) and non-germinal center type (non-GCB, most of the activated B-cell type, named ABC-type) [2]. Standard treatment is usually immune chemotherapy combined with rituximab, cyclophosphamide, doxorubicin, vincristine, and prednisone (R-CHOP). Although 50–60% of DLBCL patients can be cured by R-CHOP, the outcome of 40–50% of patients who still have relapsed/refractory (R/R) DLBCL remains poor [3]. Although understanding of the genetic and molecular landscape of DLBCL has increased significantly over the last two decades, there has been limited progress with regard to implementing this knowledge as improved upfront therapies. Recently, increasing attention has focused on the addition of various drugs to improve outcomes.

Lenalidomide is an immunomodulatory agent that is a derivative of thalidomide with fewer side effects, e.g., myelosuppression, which can limit lenalidomide's usage. Preclinical studies have shown that the antineoplastic effects of lenalidomide include direct antineoplastic activity, immunologic effects mediated by inhibition of tumor cell proliferation and angiogenesis, and stimulation of cytotoxicity mediated by T cells and NK cells [4–7]. Moreover, its activity has been demonstrated in a wide spectrum of hematologic malignancies, including myelodysplastic syndromes [8], multiple myeloma [9, 10], and B-cell NHL [11]. Several clinical trials have shown that lenalidomide has efficacy against DLBCL and is well tolerated, and it is expected to become a new treatment option for DLBCL [12–14]. Long-term follow-up combined analysis from two phase II trials showed that the efficacy of lenalidomide combined with R-CHOP (R2CHOP) was maintained over time, with a high rate of progression-free survival (PFS), and overall survival (OS); late toxicity was also low. Furthermore, considering the patients with high-risk features who were included, addition of lenalidomide to R-CHOP appears to mitigate the negative prognostic impact of the non-GCB phenotype [15]. Based on real-world data, lenalidomide plus rituximab may serve as a salvage therapy for R/R DLBCL, with a complete response rate (CRR) of 21% and an overall response rate (ORR) of 38%; the median posttreatment OS and PFS were 7.3 and 1.8 months, respectively [16]. We performed this meta-analysis to comprehensively analyze the efficacy and safety of lenalidomide in DLBCL.

## Materials and methods

### Search strategy

PubMed, Cochrane Library, and Embase were searched up to March 2022 for potential eligible published studies. We used the following search terms: [(revlimid) OR (lenalidomide)] AND (diffuse large B-cell lymphoma).

### Selection criteria

Studies were included if the following inclusion criteria were met: (a) patients: all patients diagnosed with DLBCL; (b) intervention: treatment including lenalidomide; (c) control: treatment not including lenalidomide; and (d) outcomes: primary outcomes of OS, PFS and event-free survival (EFS) and secondary outcomes of the response rate and any potential hematological adverse events (AEs); (e) study design: all included studies with a randomized controlled trial (RCT) design aiming to investigate the efficacy and safety of lenalidomide in DLBCL. The following types of articles were excluded: case reports/case series, conference abstracts/papers, reviews and meta-analyses, preclinical research, notes/letters/short surveys/editorial/comment/brief communication, retrospective/observational studies, single arm studies and studies not providing information about the effectiveness of lenalidomide in DLBCL.

### Data collection and quality assessment

The Preferred Reporting Items for Systematic Reviews and Meta-Analyses (PRISMA) statement [17] was used as a guide and template for every step of this study. The quality of the evidence was assessed using the Joanna Briggs Institute (JBI) reviewers' manual for RCTs and quasi-experimental studies [18]. The evidence level of the RCTs was level 1. The following items were extracted among treated patients from each study: authors, publication year, country, sample size, median age, sex ratio, disease status, enrollment period, phase, response rate and survival. The data extraction was conducted independently by two authors. Information was examined and adjudicated independently by an additional author referring to the original studies.

### Statistical analysis

Statistical heterogeneity was assessed using Cochran Q statistics and *I*^2^ statistics, with *I*^2^ statistics categorized as low (*I*^2^ ≤ 25%), moderate (*I*^2^ ≤ 50%), high (*I*^2^ ≤ 75%), or considerable (*I*^2^ > 75%) heterogeneity. If there was significant heterogeneity between studies (*P* < 0.10 or *I*^2^ > 50%), the random effects model was used; otherwise, the fixed effects model was chosen. A meta-analysis of proportions with 95% confidence interval (95%CI) was conducted after the data were transformed by Freeman-Tukey double arcsine transformation. The pooled hazard ratio (HR) and its 95%CI were used to evaluate survival in relation to lenalidomide in DLBCL. The pooled relative risk (RR) with 95%CI was used to assess the response rate and grade ≥ 3 hematological toxicity. Egger’s linear regression test and Begg & Mazumdar’s rank correlation tests were performed to detect publication bias. Visual inspection of funnel plot was conducted. Sensitivity analysis was conducted by sequential omission of each included study. All analyses were performed using R 4.1.1, and *P* < 0.05 was considered statistically significant for all included studies.

## Results

### Study selection and characteristics

**Fig. 1:**
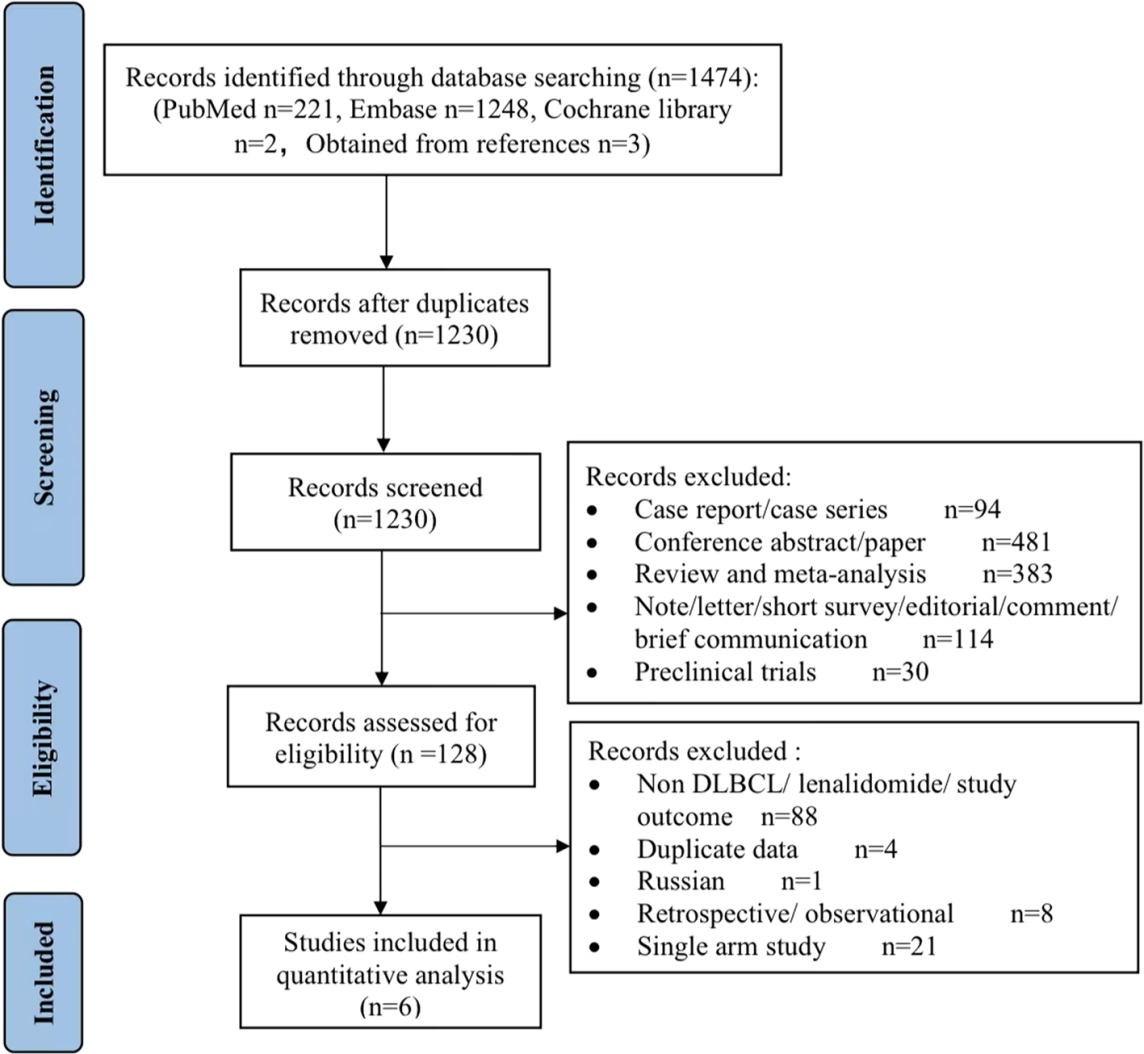
Literature search and selection

Our initial literature search yielded 1474 studies. After duplicates were removed, 1230 articles remained. A total of 1102 studies were excluded due to irrelevance after screening. The remaining 128 studies were retrieved for eligibility, 88 were excluded due to non-DLBCL/lenalidomide/study outcomes, 8 studies were retrospective/observational studies, 1 study was in Russian, 4 studies involved duplicate data, and 21 studies were single-arm studies. Eventually, 6 randomized controlled trials including 1938 patients were included in the present meta-analysis (Fig. 1) [19–24]. Information related to the population characteristics, and trial-reported results was summarized in Table 1. Among the included patients, 4 included untreated patients, and 2 included R/R cases. There were 2 phase II studies, 1 phase II/III study, and 3 phase III studies. The studies were published between 2017 and 2021 and were mainly initiated by researchers in Europe and America. The sample sizes ranged from 39 to 645. The median age of most patients was greater than 65 years, with the oldest being over 80 years.

**Table 1:** Characteristics of the included trials

Author	Year	Country	Study	Disease status	Enrollment period	Phase	Median follow-up (months)	Lenalidomide/control group		
								Regimen	Median age (range)	Female/Male
Czuczman et al. [19]	2017	America	DLC-001	R/R	–	II/III	–	LEN	69 (28–84)	21/30
								Investigator’s Choice	65 (20–84)	20/31
Thieblemont et al. [20]	2017	France	REMARC	Untreated	2009.05–2014.05	III	52	LEN	69 (58–80)	140/183
								Placebo	68 (59–80)	147/180
Kühnl et al. [21]	2020	UK	LEGEND	R/R	2013.10–2016.11	II	21.5 for living pts	LEN+R-GEM	58 (21–75)	8/13
								R-GEM-P	59 (21–77)	5/14
Nowakowski et al. [22]	2021	America	ECOG-ACRIN E1412	Untreated	2013.08–2017.01	II	36	LEN+R-CHOP	67 (24–88)	51/94
								R-CHOP	66 (37–92)	59/76
Oberic et al. [23]	2021	France	SENIOR	Untreated	2014.08–2017.09	III	25.1	LEN+R-MiniCHOP	≥ 80	65/57
								R-MiniCHOP	≥80	71/56
Nowakowski et al. [24]	2021	America	ROBUST	Untreated	2015.02–2017.08	III	27.1 (0–47) for living pts	LEN+R-CHOP	65 (21–82)	121/164
								Placebo+R-CHOP	65 (28–83)	142/143

*R/R* relapsed/refractory, *LEN* lenalidomide, *pts*: patients, *R-CHOP* rituximab, cyclophosphamide, doxorubicin, vincristine, and prednisone, *R-GEM* rituximab, methylprednisolone and gemcitabine, *R-GEM-P* rituximab, methylprednisolone, gemcitabine, cisplatin, *R-MiniCHOP* standard attenuated dose of R-CHOP

### Response rate

**Fig. 2:**
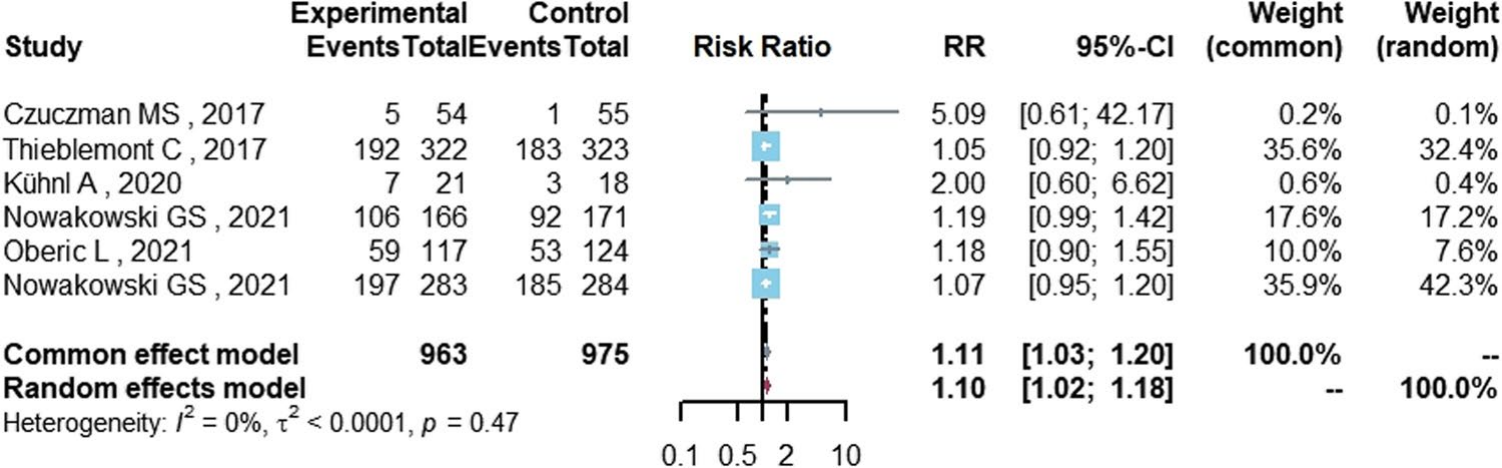
The forest plot of CRR

Among 963 DLBCL patients in the group containing lenalidomide, ORR was 67% (95%CI 45.7–85.3%), CRR was 47.7% (95%CI 28.5–67.2%), and the partial response rate (PRR) was 16.3% (95%CI 10.6–23.0%). In the control group without lenalidomide, which included 975 DLBCL patients, the ORR was 56.9% (95%CI 31.4–80.6%), the CRR was 37.8% (95%CI 16.7–61.5%), and the PRR was 15.6% (95%CI 10.1–21.9%). The CRR in the lenalidomide group was significantly higher than that in the control group (RR = 1.11, 95%CI 1.03–1.20, *P* = 0.008) (Table 2, Fig. 2). Statistical significance was not found for ORR or PRR.

**Table 2:** Response rate and safety of lenalidomide in DLBCL

Primary outcomes	Lenalidomide group		Control group		No of studies	No. of patients	*I*^2^ (%)	P value for heterogeneity	RR (95%CI)	*P* value for effects model
	Pooled rate (%, 95%CI)	*I*^2^ (%)	Pooled rate (%, 95%CI)	*I*^2^ (%)						
*Response rate*										
ORR	67.0 (45.7, 85.3)	96.5	56.9 (31.4, 80.6)	97.5	6	1938	61.2	0.024	1.09 (0.99, 1.20)	0.080
CRR	47.7 (28.5, 67.2)	94.5	37.8 (16.7, 61.5)	96.1	6	1938	0	0.468	1.11 (1.03, 1.20)	*0.008*
PRR	16.3 (10.6, 23.0)	87.7	15.6 (10.1, 21.9)	85.7	6	1938	0	0.423	0.96 (0.79; 1.17)	0.674
*Safety*										
Neutropenia	48.3 (37.5, 59.1)	86.6	31.7 (19.7, 44.9)	94.6	6	1938	86.0	< 0.001	1.56 (1.15, 2.11)	*0.004*
Thrombocytopenia	13.7 (5.7, 24.2)	95.2	10.5 (1.9, 24.1)	94.8	6	1938	75.5	0.001	1.55 (0.71, 3.37)	0.272
Anemia	17.3 (9.9, 26.1)	81.7	16.0 (8.3, 25.5)	82.8	5	1293	53.9	0.070	1.21 (0.79, 1.87)	0.383
Febrile neutropenia	11.9 (5.2, 20.6)	87.1	5.8 (1.8, 11.5)	81.7	5	1293	0	0.891	1.81 (1.31, 2.49)	< *0.001*

Italic values indicate *P* < 0.05

*ORR* overall response rate, *CRR* complete response rate, *PRR* partial response rate, *95%CI* 95% confidence interval, *RR* relative risk

### PFS, EFS and OS

**Fig. 3:**
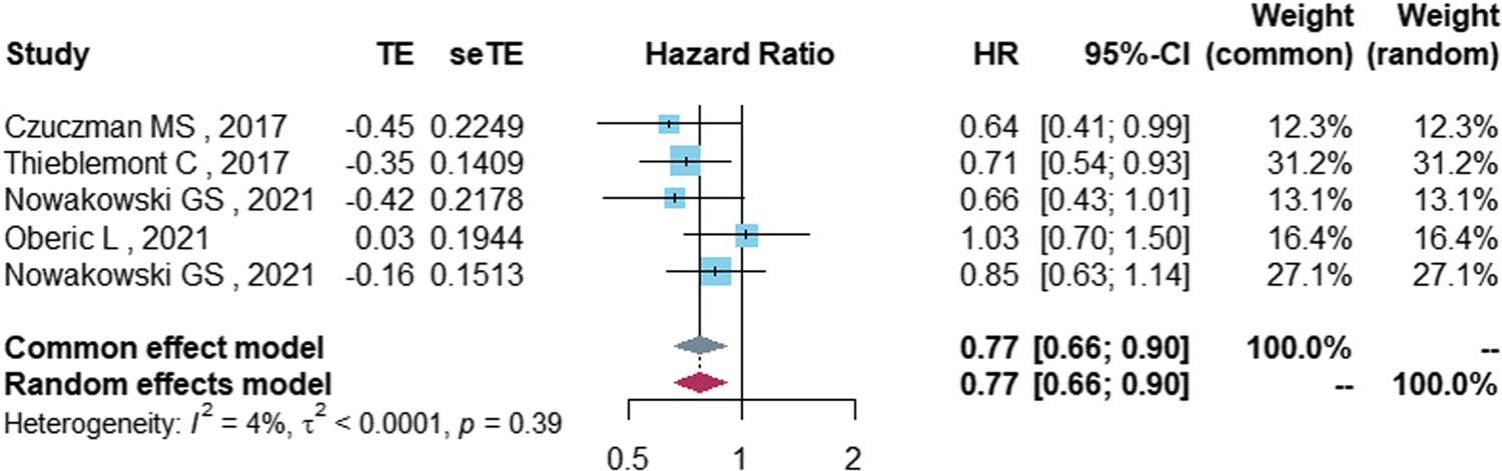
The forest plot of PFS

Regarding meta-analysis evaluating survival, five studies with 1899 patients analyzed the PFS of DLBCL patients treated with lenalidomide. Low heterogeneity was found among the included studies (*I*^2^ = 3.6%). The overall estimation in the fixed effects model showed a PFS benefit in favor of the control group not treated with lenalidomide (HR = 0.77, 95%CI 0.66–0.90, *P* = 0.001) (Fig. 3, Table 3). Subgroup analysis showed a survival benefit in the untreated (HR = 0.79, 95%CI 0.67–0.94, *P* = 0.006), R-CHOP-based (HR = 0.75, 95%CI 0.62–0.90, *P* = 0.002), and ≥ 65-year-old (HR = 0.77, 95%CI 0.66–0.90, *P* = 0.001) populations. There was no significant benefit in GCB (HR = 0.70, 95%CI 0.48–1.03, *P* = 0.070) or non-GCB (HR = 0.83, 95%CI 0.66–1.05, *P* = 0.125) patients.

**Table 3:** Survival analysis of lenalidomide in DLBCL

Secondary outcomes	No. of studies	No. of patients	*I*^2^ (%)	*P* value for heterogeneity	HR (95%CI)	*P* value for effects model
*PFS*						
ALL	5	1899	3.6	0.386	0.77 (0.66, 0.90)	*0.001*
Disease status						
Untreated	4	1790	10.1	0.343	0.79 (0.67, 0.94)	*0.006*
Regimen						
R-CHOP based	3	1549	0	0.550	0.75 (0.62, 0.90)	*0.002*
Median age						
≥ 65	5	1899	3.6	0.386	0.77 (0.66, 0.90)	*0.001*
Subtype						
Non-GCB	3	834	47.6	0.148	0.83(0.66, 1.05)	0.125
GCB	3	350	0	0.484	0.70 (0.48, 1.03)	0.070
*OS*						
ALL	5	1601	0	0.592	0.99 (0.83, 1.20)	0.950
*EFS*						
ALL	3	847	0	0.818	0.99 (0.81, 1.21)	0.927

Italic values indicate *P* < 0.05

*EFS* event-free survival, *PFS* progression-free survival, *OS* overall survival, *95%CI* 95% confidence interval, *HR* hazard ratio, *R-CHOP* rituximab, cyclophosphamide, doxorubicin, vincristine, and prednisone, *GCB* germinal center type

Also, 1601 DLBCL patients from five trials were available for analysis of OS, with 847 patients from 3 trials for EFS. The estimation of OS and EFS were similar in the two groups, with pooled HRs of 0.99 (95%CI: 0.83–1.20, *I*^2^ = 0, *P* = 0.950) and 0.99 (95%CI: 0.81–1.21, *I*^2^ = 0, *P* = 0.927), respectively.

### Safety analysis

**Fig. 4:**
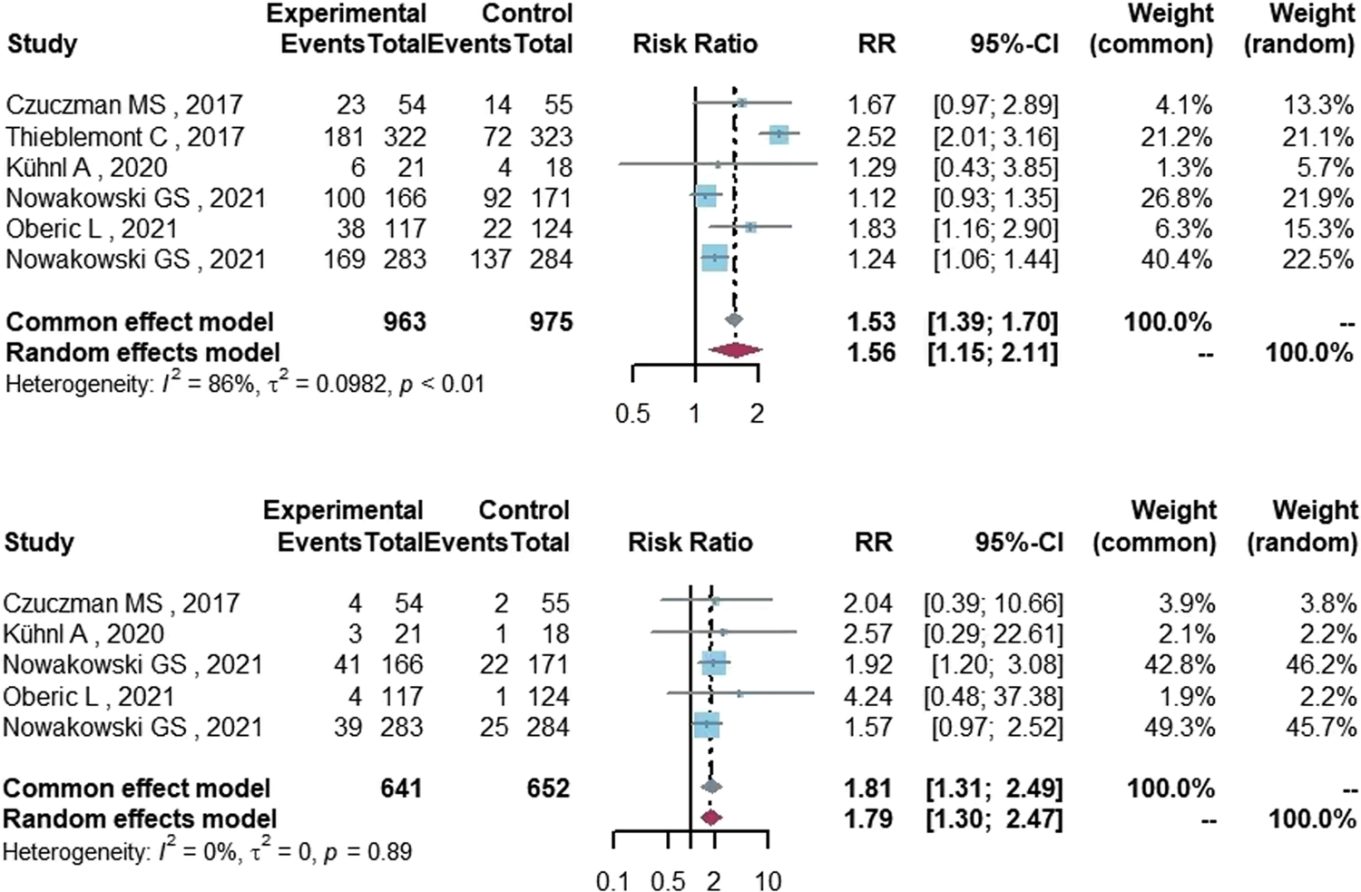
The forest plot of neutropenia and febrile neutropenia

As shown in Table 2, the incidence of neutropenia in the lenalidomide group (48.3%, 95%CI 37.5–59.1%) was higher than that of anemia (17.3%, 95%CI 9.9–26.1%), thrombocytopenia (13.7%, 95%CI 5.7–24.2%) and febrile neutropenia (11.9%, 95%CI 5.2–20.6%). The lenalidomide group had a significant incidence of grade ≥ 3 hematological AEs involving neutropenia (RR = 1.56, 95%CI 1.15–2.11, *P* = 0.004, Fig. 4) and febrile neutropenia (RR = 1.81, 95%CI 1.31–2.49, *P* < 0.001, Fig. 4). The incidence of anemia and thrombocytopenia was similar between the lenalidomide group and the control group (RR = 1.21, 95%CI 0.79–1.87, *P* = 0.383; RR = 1.55, 95%CI 0.71–3.37, *P* = 0.272, respectively).

### Sensitivity analysis

**Fig. 5:**
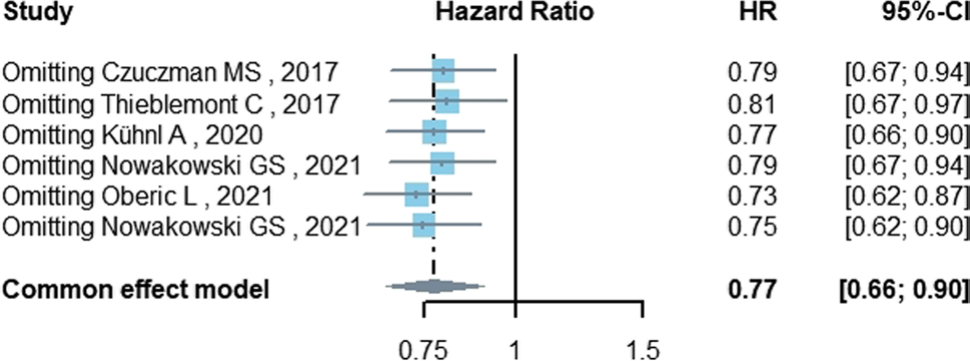
Sensitivity analysis for PFS

Sensitivity analysis was conducted by omitting one study at a time and analyzing the remaining studies. The results are shown in Fig. 5, with no substantial changes, showing the reliability and stability of our results.

### Publication bias

**Fig. 6:**
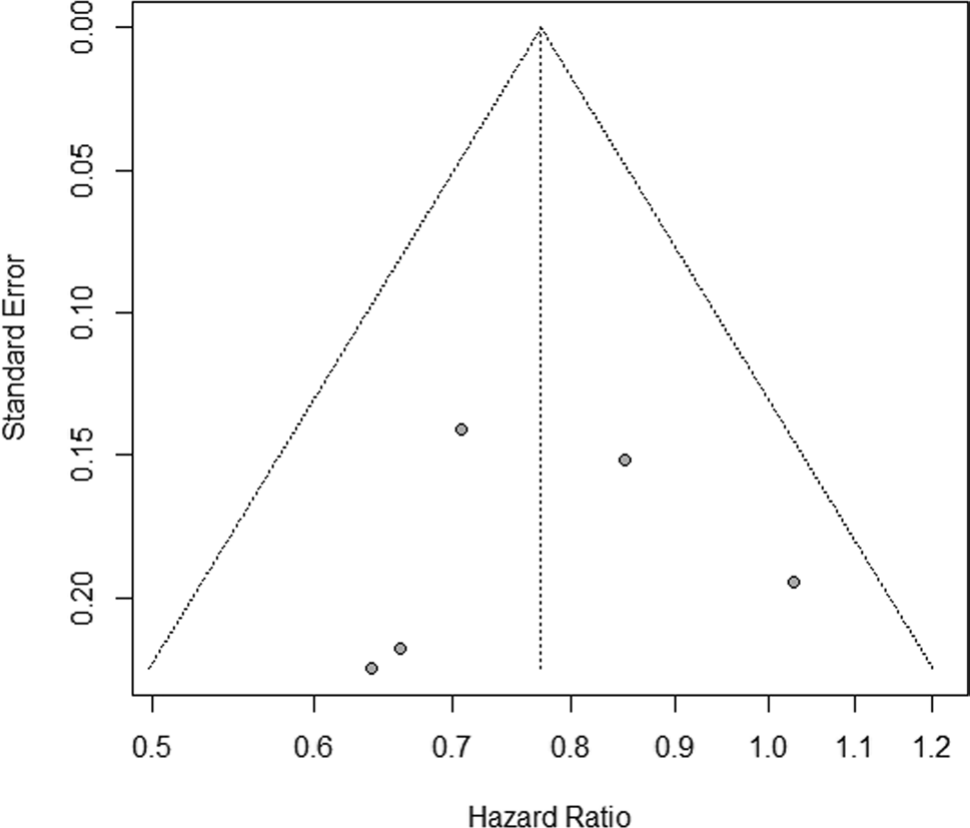
Publication bias based on funnel plot

Based on the results of Begg & Mazumdar's (*P* = 0.327) and Egger's (*P* = 0.809) tests, there was no significant publication bias (Fig. 6).

## Discussion

Lenalidomide’s antineoplastic effects have shown a good synergistic effect when combined with anti-CD20 monoclonal antibodies, as the agent enhances the NK-cell and antibody-dependent cell-mediated cytotoxicity of the anti-CD20 monoclonal antibody [25, 26]. The efficacy and safety of lenalidomide have been investigated extensively since the Mayo Clinic first reported a phase I study in which lenalidomide was combined with R-CHOP as front-line treatment in DLBCL patients and safely combined with R-CHOP without affecting the dose intensity of chemoimmunotherapy [27].

The phase II MC078E study showed that lenalidomide in combination with standard frontline treatment R-CHOP produced high response rates; the ORR in the intent-to-treat population was 97% (32/33), 29 (88%) had CR, and 3 had PR [28]. In a phase I study of lenalidomide plus R-CHOP, the ORR was 90%, with 81% of untreated, elderly patients with DLBCL achieving CR [29]. The CRR and ORR of lenalidomide in combination with R-ESHAP (rituximab, etoposide, cisplatin, cytarabine, methylprednisolone) in patients with R/R DLBCL were reported to be 47.4% and 78.9%, respectively [30]. As a second-line treatment for DLBCL, 38.9% of patients achieved CR with R-GEM-L (rituximab, methylprednisolone gemcitabine, and lenalidomide) [21]. The ORR for lenalidomide monotherapy in R/R patients was 33.3% [31]. Lenalidomide plus ibrutinib and rituximab have promising activity in R/R DLBCL, with an ORR of 44% (CRR, 28%) [32]. Dual translocation of MYC and BCL2 in patients with DLBCL is termed “double-hit lymphoma” (DHL), and dual protein overexpression of MYC and BCL2 without underlying translocations is termed “double-expressor lymphoma” (DEL). Both DHL and DEL are recognized as a distinct subset of non-Hodgkin lymphoma that is associated with very poor outcomes [33–35]. The combination of lenalidomide with dose-adjusted (DA)-EPOCH-R (etoposide, prednisone, vincristine, cyclophosphamide, doxorubicin, and rituximab) for DLBCL treatment-naive patients shows evidence of DHL or DEL. The best responses after induction were 13 complete responses (87%) and 1 partial response (7%), with 1 case of progressive disease (7%) [36]. Among the included studies, the SENIOR study presented the proportion of patients with expression or rearrangement of MYC and BCL2, and the ORR at the end of treatment was 73% in the R-miniCHOP arm and 82% in the R2-miniCHOP arm [23]. And the vast majority of patients were newly diagnosed and treated with R-CHOP/R-miniCHOP. The ORR in the lenalidomide group was 67%, the CRR 47.7%, and the PRR 16.3% among 963 DLBCL patients, higher than in the control group. In the control group, the ORR was 56.9%, the CRR was 37.8%, and the PRR was 15.6%. However, only the CRR was significantly higher in the lenalidomide group than in the control group.

The prognosis of elderly patients with newly diagnosed DLBCL is worse than that of young patients. Comorbidities and physiological organ function impairment often result in unmanageable toxicities and limit optimal chemotherapy. Our quantitative analysis showed that addition of lenalidomide resulted in a statistically significant improvement in PFS but failed to improve OS and EFS. Subgroup analysis showed survival benefits in the untreated, R-CHOP-treated, and ≥ 65-year-old populations. In the ECOG-ACRIN E1412 study with a median age of 66 years old [22], R2CHOP was associated with a 34% reduction in the risk of progression or death compared with R-CHOP. The 1-, 2-, and 3-year PFS rates were 84% versus 73%, 76% versus 69%, and 73% versus 62% for R2CHOP versus R-CHOP, respectively. The phase III REMARC study showed that lenalidomide maintenance for 24 months after obtaining CR or PR with R-CHOP significantly prolonged PFS in untreated elderly patients with DLBCL. The 2-year PFS was improved from 75% (95%CI 70–80%) to 80% (95%CI 75–84%) in the lenalidomide group [20]. These results were similar to our results.

It is well known that the prognosis of non-GCB is worse than that of GCB in the R-CHOP era [37, 38]. An increasing number of studies have also shown that lenalidomide combined with R-CHOP overcomes the negative impact of the non-GCB phenotype in untreated DLBCL and has promising clinical activity in DLBCL [15]. A retrospectively assessed 123 R/R DLBCL patients showed that lenalidomide is more efficient in non-GCB DLBCL, with complete remission was achieved in 32% and a partial remission in 33% non-GCB patients compared with 0% and 3% in the GCB group [39]. In a phase II trial, the addition of lenalidomide appears to mitigate a negative impact of non-GCB phenotype on patient outcome [40]. There was no significant benefit in either GCB or non-GCB patients in our study. The possible reason is the different typing methods based on Hans and gene expression profiling (GEP) among the included trials. Alternatively, more cases may be needed.

The addition of a new drug to chemoimmunotherapy raises concerns about increased toxicity, especially in older patients. Wang M et al. [41] reported common grades 3–4 hematological adverse events (≥ 10 events), including neutropenia (53%), lymphopenia (40%), thrombocytopenia (33%), leukopenia (27%) and anemia (18%). Ferreri et al. [42] found lenalidomide was well tolerated, especially in this elderly population, with the exception of neutropenia, grade-4 toxicities occurred in < 1% of courses. Our study summarized grade ≥ 3 hematological toxicity events. The results show that the pooled incidence of neutropenia was higher than that of thrombocytopenia, anemia, and febrile neutropenia. Compared to the control group without lenalidomide, the lenalidomide group had a significant incidence of grade ≥ 3 hematological AEs involving neutropenia and febrile neutropenia.

This study has several limitations. First, the results may be affected by heterogeneity caused by many factors, such as different inclusion criteria for the individual studies, inconsistent induction therapy. Second, some stratified analyses according to study or patient characteristics were not performed because several treatments were reported without more information. Therefore, the results should be considered cautiously. Further investigation is essential to provide reliable proof.

In conclusion, DLBCL patients treated with lenalidomide have a higher CRR, resulting in better PFS but a higher incidence of grade ≥ 3 hematological AEs involving neutropenia and febrile neutropenia.
